# Spectral imaging with deep learning

**DOI:** 10.1038/s41377-022-00743-6

**Published:** 2022-03-16

**Authors:** Longqian Huang, Ruichen Luo, Xu Liu, Xiang Hao

**Affiliations:** 1 State Key Laboratory of Modern Optical Instrumentation, College of Optical Science and Technology, Zhejiang University, Hangzhou, 310027 China; 2 College of Computer Science and Technology, Zhejiang University, Hangzhou, 310027 China; 3 Jiaxing Key Laboratory of Photonic Sensing & Intelligent Imaging, Jiaxing, 314000 China; 4 Intelligent Optics & Photonics Research Center, Jiaxing Research Institute Zhejiang University, Jiaxing, 314000 China

**Keywords:** Imaging and sensing, Optical spectroscopy

## Abstract

The goal of spectral imaging is to capture the spectral signature of a target. Traditional scanning method for spectral imaging suffers from large system volume and low image acquisition speed for large scenes. In contrast, computational spectral imaging methods have resorted to computation power for reduced system volume, but still endure long computation time for iterative spectral reconstructions. Recently, deep learning techniques are introduced into computational spectral imaging, witnessing fast reconstruction speed, great reconstruction quality, and the potential to drastically reduce the system volume. In this article, we review state-of-the-art deep-learning-empowered computational spectral imaging methods. They are further divided into amplitude-coded, phase-coded, and wavelength-coded methods, based on different light properties used for encoding. To boost future researches, we’ve also organized publicly available spectral datasets.

## Abstract (web summary)

This review categorizes deep-learning-based computational spectral imaging methods and provides insight into amplitude, phase, and wavelength-based light encoding strategies for deep-learning spectral reconstruction.

## Graphical abstract

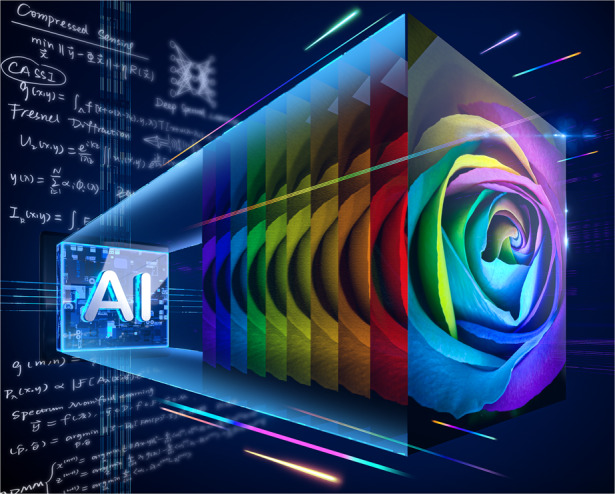

## Introduction

**Fig. 1:**
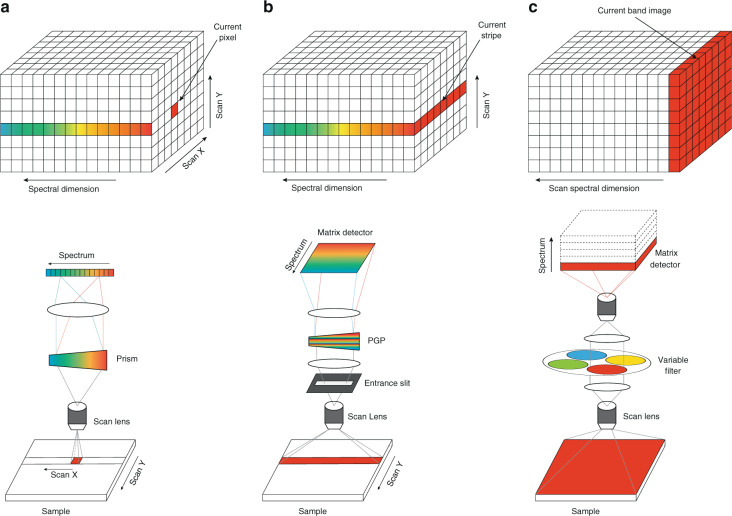
Typical scanning spectral imaging approaches. **a** Whiskbroom. **b** Pushbroom. PGP prism-grating-prism. **c** Wavelength scan

With the ability of getting distinctive information in spatial and spectral domain, spectral imaging technology has vast applications in remote sensing^1^, medical diagnosis^2^, biomedical engineering^3^, archeology and art conservation^4^, and food inspection^5^. Traditional methods of spectral imaging include whiskbroom scanning, pushbroom scanning, and wavelength scanning. Whiskbroom spectroscopy performs scanning pixel by pixel. A widely acknowledged example is Airborne Visible/Infrared Imaging Spectrometer^6,7^, which implemented whiskbroom approach on aircraft for Earth remote sensing. Pushbroom scan system uses the entrance slit and builds image line by line. The Hyperspectral Digital Imagery Collection Experiment instrument^8,9^ implemented pushbroom imaging optics with a prism spectrometer, offering a good capability for remote sensing. Wavelength scanning methods capture spectral image cubes through swapping narrow bandpass filters in front of the camera lens or using electronically tunable filters^10,11^. These typical scanning spectral imaging approaches are illustrated in Fig. 1.

However, traditional scanning methods suffer from the low speed of the spectral image acquisition process because of the time-consuming scanning mechanism. As a consequence, they are not applicable for large scenes or dynamic recording. To solve this problem, researchers started to explore snapshot spectral imaging methods^12^. Early endeavors include integral field spectrometry, multispectral beam splitting, and image-replicating imaging spectrometer, as mentioned in ref. ^12^. These methods cannot obtain massive spectral channels and have bulky optical systems, though achieving multispectral imaging through splitting light.

With the development of compressed-sensing (CS) theory^13,14^, compressive spectral imaging has received growing attention from researchers because of its elegant combination of optics, mathematics and optimization theory. It has the ability to perform spectral imaging through fewer measurements, which is essential in resource-constrained environments. Compressive spectral imaging techniques often use a coded aperture to block or filter the input light field, namely the encoding process in the compressive sensing pipeline. As the name indicates, this process plays a role in information compression, which is flexible in design and provides the prior knowledge for later reconstruction. Different from the hardware-based encoding, its decoding process requires the computation via designed algorithms. Traditional reconstruction approach is iterative, using designed measurement of the encoding process and other prior knowledge for reconstruction. As a consequence, the decoding procedure is computationally expensive and can take minutes or even hours for spectral reconstruction. Furthermore, degradation problem when using fewer measurements also limits its application in resource-constrained environments.

While using coded aperture for amplitude encoding has shown the capability of spectral imaging from fewer measurements, the reduced light throughput and large system volume make it unsuitable for practical applications. To overcome this drawback, phase-coded spectral imaging^15,16^ is developed to improve light throughput and reduce system volume. Its main idea is using a carefully-designed thin diffraction optical element to manipulate the input light phase, which will affect the spectra in the diffraction process. Then, to recover spectra modeled in the complex diffraction process, powerful deep-learning techniques are required.

Researchers in computer graphics are also seeking to optimize spectra reconstruction, because using spectra is better than RGB triplets when rendering a scene illumination or display a virtual object on a monitor device. Early works^17–19^ obtain spectrum from RGB triplet, but this can be an ill-posed problem that has non-unique solutions and negative spectrum values. Later works involved more effective methods such as basis function fitting^20^ and dictionary learning^21^. The latter is based on the hyperspectral dataset, yet still have the problem of long-time weight fitting procedure. As demonstrated in a statistical research on hyperspectral images^22^, spectra within an image patch are correlated. Nevertheless, these pixel-wise methods fail to exploit the correlation information in a spectral data cube, hence effective patch feature extraction algorithms are expected. The pursuit of accurate and fast RGB-to-spectra approach has pushed the development of wavelength-coded methods. Researchers extended the RGB filters to multiple self-designed broadband filters for delicate wavelength encoding, and a reliable decoding algorithm is in demand. Completing such complex computing tasks is the mission of deep learning.

To alleviate the high computation costs in the aforementioned methods, deep-learning algorithm has been proposed as an alternative for learning spatial–spectral prior and spectral reconstruction. Deep-learning techniques can perform faster and more accurate reconstruction than iterative approaches, thus is suitable to apply on spectral recovery tasks. In recent years, many works have employed deep-learning models (such as convolutional neural networks, CNNs) in their spectral imaging framework and showed improved reconstruction speed and quality^15,16,23–25^.

In this review, we will look back at the development in spectral imaging with deep-learning tools and look forward to the future directions for computational spectral imaging systems with deep-learning technology. In the following sections, we will first discuss the deep-learning-empowered compressive spectral imaging methods that perform amplitude encoding using coded apertures in “Amplitude-coded Spectral Imaging”. We will then introduce phase-coded methods that use diffractive optical element (DOE, or diffuser) in “Phase-coded Spectral Imaging”. In “Wavelength-coded Spectral Imaging”, we will introduce wavelength-coded methods that use RGB or broadband optical filters for wavelength encoding, and adopt deep neural networks for spectral reconstruction. To boost future researches on learned spectral imaging, we have organized existing spectral datasets and the evaluation metrics (in “Spectral Imaging Datasets”). Finally, we will summarize the deep-learning-empowered spectral imaging methods in “Conclusions and Future Directions” and share our thoughts on the future.

## Amplitude-coded spectral imaging

**Fig. 2:**
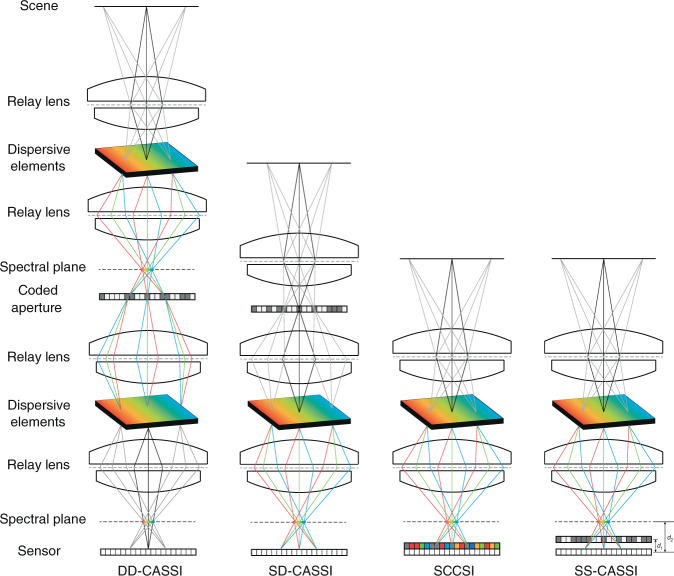
Illustration of four CASSI architectures. In DD-CASSI architecture, the spectral scene experiences a shearing-coding-unshearing procedure. In SD-CASSI, the diffraction grating before the coded aperture is removed, therefore it becomes a coding-shearing process. In SCCSI, the coded aperture is placed behind the dispersive element and the spectral data will experience a shearing-coding procedure. In SS-CASSI, coded aperture position becomes flexible between spectral plane and camera sensor, where the ratio (*d*_1_ + *d*_2_)/*d*_2_ determines the extent of spectral encoding

Amplitude-coded methods use coded aperture and dispersive elements for compressive spectral imaging. The classical system is coded aperture snapshot spectral imager (CASSI). To date, there are four CASSI architectures based on different spatial–spectral modulation styles, as shown in Fig. 2. The first proposed architecture is dual-disperser CASSI (DD-CASSI)^26^, which consists of two dispersive elements for spectral shearing with a coded aperture in between. Single-dispersive CASSI (SD-CASSI)^27^ is a later work, using one dispersive element placed behind the coded aperture. Snapshot colored compressive spectral imager (SCCSI)^28^ uses also a coded aperture and a dispersive element, but places the coded aperture behind the dispersive element. In comparison to SCCSI that attaches colored coded aperture (or, color filter array) to the camera sensor, spatial–spectral CASSI (SS-CASSI) architecture^29^ adds the flexibility of coded aperture position between spectral plane and sensor plane. This increases the complexity of the coded-aperture model, which may play a role in improving the system performance. Some deep-learning-based compressive spectral imaging methods have found better results with SS-CASSI^30,31^.

### Coded-aperture model

Since most works were based on SD-CASSI system, we will give a detailed derivation of the image construction process of SD-CASSI. The image formation procedure is different for other CASSI architectures in Fig. 2, but the key processes (vectorization, discretization, etc.) are the same. We refer readers to refs. ^26,28,29^ for a detailed modeling of the DD-CASSI, SCCSI, and SS-CASSI, respectively.

**Fig. 3:**
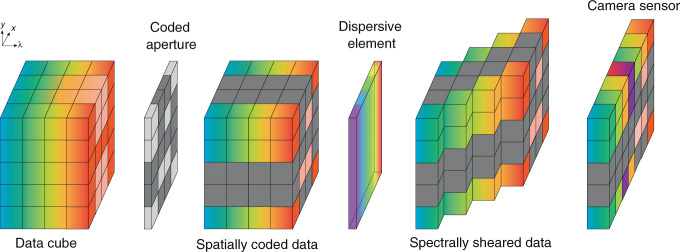
Spectral imaging process within SD-CASSI architecture. The spectral data cube first passes a coded aperture for spatial encoding, then its spectral arrangement is shifted by a dispersive element. Finally, a detector captures the spatial and spectral encoded data image

At the time when SD-CASSI was proposed, coded aperture had block–unblock pattern, which was extended to colored pattern in ref. ^32^. We will use a colored coded aperture in derivation for generality. Consider a target scene with spectral density *f*(*x*, *y*, *λ*) and track its route in an SD-CASSI system: it first encounters a coded aperture with transmittance *T*(*x*, *y*, *λ*) and then is sheared by a dispersive element (assume at *x*-axis), finally punches on the detector array. Figure 3 illustrates the whole process.

The spectral density before the detector is formulated as

1 $$\begin{array}{lll}g(x,y,\lambda )&=&\iint \delta \left(x^{\prime} \,-\,[x\,+\,\alpha (\lambda \,-\,{\lambda }_{c})]\right)\delta (y^{\prime} \,-\,y)\,\cdot\, f(x^{\prime} ,y^{\prime} ,\lambda )\,T(x^{\prime} ,y^{\prime} ,\lambda )\,\,{{\mbox{d}}}x^{\prime} {{\mbox{d}}}\,y^{\prime} \\ &=&f(x\,+\,\alpha (\lambda \,-\,{\lambda }_{c}),y,\lambda )\,T(x\,+\,\alpha (\lambda \,-\,{\lambda }_{c}),y,\lambda )\end{array}$$

where delta function represents the spectral dispersion introduced by the dispersive element, such as a prism or gratings. *α* is a calibration factor, and *λ*_*c*_ is the center wavelength of dispersion. Since we can only measure the intensity on the detector, the measurement should be the integral along the wavelength:

2 $$\begin{array}{lll}g(x,y)&=&\int _{{{\Lambda }}}g(x,y,\lambda )\,\,{{\mbox{d}}}\,\lambda \\ &=&\int _{{{\Lambda }}}f(x\,+\,\alpha (\lambda \,-\,{\lambda }_{c}),y,\lambda )\,T(x\,+\,\alpha (\lambda -{\lambda }_{c}),y,\lambda )\,\,{{\mbox{d}}}\,\lambda \end{array}$$

where Λ is the spectrum range.

Next, we discretize Eq. (2). Denote Δ as the pixel size (in *x* and *y* dimension) of the detector, and assume the coded aperture has square pixel size Δ_code_ = *q*Δ, *q* ≥ 1. The code pattern is then represented as a spatial array of its pixels:

3 $$T(x,y,\lambda )\,=\,\mathop{\sum}\limits_{m,n}T(m,n,\lambda ){{{\rm{rect}}}}\left(\frac{x}{q{{\Delta }}}\,-\,m,\frac{y}{q{{\Delta }}}\,-\,n\right)$$

Finally, signals within the region of a pixel will be accumulated in the sampling process:

4 $$\begin{array}{ll}g(m,n)&=\iint g(x,y){{{\rm{rect}}}}\left(\frac{x}{{{\Delta }}}\,-\,m,\frac{y}{{{\Delta }}}\,-\,n\right)\,{{{\rm{d}}}}x{{{\rm{d}}}}y\\ &=\iint {{{\rm{d}}}}x{{{\rm{d}}}}y\,{{{\rm{rect}}}}\left(\frac{x}{{{\Delta }}}\,-\,m,\frac{y}{{{\Delta }}}\,-\,n\right)\int _{{{\Lambda }}}{{{\rm{d}}}}\lambda \,f\left(x\,+\,\alpha (\lambda \,-\,{\lambda }_{C}),y,\lambda \right)\\ &\qquad\times \left[\mathop{\sum}\limits_{m^{\prime} ,n^{\prime} }T(m^{\prime} ,n^{\prime} ,\lambda ){{{\rm{rect}}}}\left(\frac{x\,+\,\alpha (\lambda \,-\,{\lambda }_{C})}{q{{\Delta }}}\,-\,m^{\prime} ,\frac{y}{q{{\Delta }}}\,-\,n^{\prime} \right)\right]\end{array}$$

To further simplify Eq. (4), we discrete *f* and *T* using their central pixel intensity. Take spectral resolution Δ_*λ*_ as the spectral interval. We use the intensity *f*(*m*, *n*, *l*) ($m,n,l\in {\mathbb{N}}$) to represent a pixel of the spectral density *f*(*x*, *y*, *λ*), where *x* $\in$ [*m*Δ − Δ/2, *m*Δ + Δ/2], *y* $\in$ [*n*Δ − Δ/2, *n*Δ + Δ/2], *λ* $\in$ [*λ*_*C*_ + *l*Δ_*λ*_ − Δ_*λ*_/2, *λ*_*C*_ + *l*Δ_*λ*_ + Δ_*λ*_/2]. Adjust the calibration factor *α* so that the dispersion distance satisfies $\alpha {{{\Delta }}}_{\lambda }\,=\,k{{\Delta }},k\,\in\, {\mathbb{N}}$. Then Eq. (4) becomes

5 $$g(m,n)\,=\,\mathop{\sum}\limits_{l}f(m\,+\,lk,n,l)\,T\left(\lfloor \frac{m\,+\,lk}{q}\,+\,\frac{1}{2}\rfloor ,\lfloor \frac{n}{q}\,+\,\frac{1}{2}\rfloor \right)$$

**Fig. 4:**
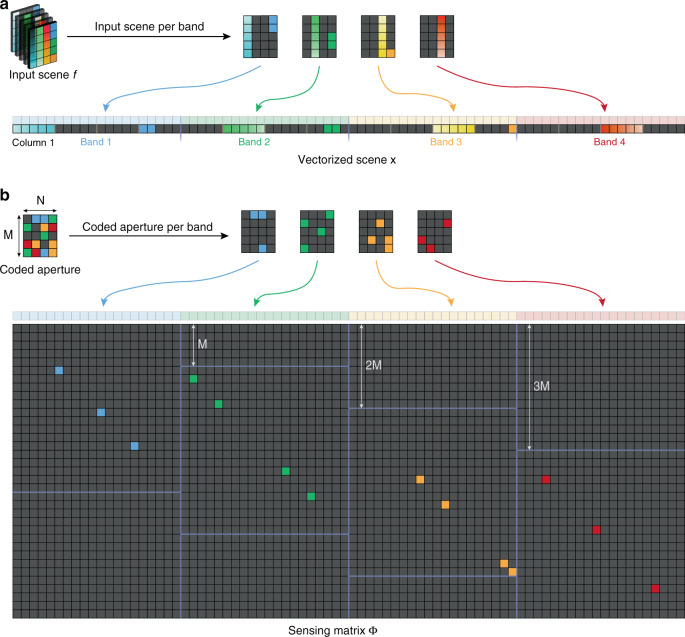
Vectorization and coded-aperture-related sensing matrix generation procedure. **a** Illustration of the vectorization process. For a matrix *A*, vectorization means stacking the columns of *A* on top of one another; For a spectral cube of the input scene *f*(*m*, *n*, *l*), vectorization means stacking the vectorized 2D slice on top of one another. **b** Illustration of generating sensing matrix from colored coded aperture in SD-CASSI architecture. It consists of a set of diagonal patterns that repeat in the horizontal direction, each time with a unit downward shift *M* that accounts for dispersion. Each diagonal pattern is generated from the vectorized coded aperture pattern of a band. The block–unblock coded aperture is similar, just turning the color bands into black and white

To adopt reconstruction algorithms, we need to rewrite Eq. (5) in a matrix form. This procedure is illustrated in Fig. 4.

First, we vectorize the measurement and spectral cube as Fig. 4a:

6 $$\begin{array}{lll}{{{\bf{y}}}}&=&{{{\rm{vect}}}}\left[g(m,n)\right],\\ {{{\bf{x}}}}&=&{{{\rm{vect}}}}\left[f(m,n,l)\right]\end{array}$$

where the measurement term $g\in {{\mathbb{R}}}^{M\,\times\, N}$ and spectral cube $f\in {{\mathbb{R}}}^{M\,\times\, N\,\times\, L}$, with spatial dimension *M* × *N* and spectral dimension *L*. After vectorization, we have the vectorized terms ${{{\bf{y}}}}\,\in\, {{\mathbb{R}}}^{MN},{{{\bf{x}}}}\,\in\, {{\mathbb{R}}}^{MNL}$.

Next, the coded aperture and dispersion shift are modeled into a sensing matrix ${{\Phi }}\,\in\, {{\mathbb{R}}}^{MV\,\times\, MNL},$ where *V* = *N* + *k*(*L* − 1) contains the dispersion shift (the shift distance is $\alpha {{{\Delta }}}_{\lambda }\,=\,k{{\Delta }},k\,\in\, {\mathbb{N}}$). A sensing matrix (for *k* = 1) produced from a colored coded aperture is shown in Fig. 4b.

Finally, the reconstruction problem is formulated as

7 $$\mathop{\min }\limits_{{{{\bf{x}}}}}\parallel {{{\bf{y}}}}\,-\,{{\Phi }}{{{\bf{x}}}}\parallel \,+\,\eta R({{{\bf{x}}}})$$

where Φ is the sensing matrix, and **y** is measurement. Term *R* stands for priority, which is a regularizer determined by the prior knowledge of the input scene **x** (e.g., sparsity), and term *η* is a weight for the prior knowledge.

### Deep compressive reconstruction

Traditional methods for spectral image reconstruction usually utilize iterative optimization algorithms, such as GAP-TV^33^, ADMM^34^, etc. These methods suffers a long reconstruction time for iterations. Besides, the spatial and spectral reconstruction accuracy is not solid by using hand-crafted priors. For example, total variance (TV) prior is always used in reconstruction algorithms, but it sometimes brings over-smoothness to the result.

Deep-learning techniques can be applied to each step in amplitude-coded spectral imaging methods, from the design of amplitude encoding strategy (coded aperture optimization) to finding a representative regularizer (term *R* in Eq. (7)), and the whole reconstruction process can be substituted with a neural network. Adopting deep-learning methods can improve the reconstruction speed by hundred of times. Moreover, learning priors from large amount of spectral data by neural networks can promote the reconstruction accuracy in both spatial and spectral domains. We have summarized recent years’ works of deep-learning-based coded aperture spectral imaging in Table 1 for comparison.

**Table 1:** Comparison of different amplitude-coded compressive spectral imaging methods

Article	CSI architecture	Performance (PSNR)	Reconstruction model	Deep-learning techniques
AutoEncoder^30^	SD/DD/SS CASSI	32.46 on CAVE (SS-CASSI)	Autoencoder Equation (Eq. (11) in ref. ^30^)	Autoencoder prior
HyperReconNet^39^	SD CASSI	33.63 on ICVL, 31.36 on Harvard	CNN	Hardware representation layer (joint training)
Spatial–spectral prior^24^	SD CASSI	34.13 on ICVL, 32.84 on Harvard, 30.03 on KAIST	Unrolled network	Learned network prior
External–internal learning^35^	SD CASSI	35.884 on ICVL, 33.585 on Harvard, 29.055 on CAVE	CNN	Dense structure, back-projection pixel loss
*λ*-Net^36^	SD CASSI	32.29 on ICVL (average of 16 scenes)	conditional GAN	Self-attention, hierarchical structure
DNU^42^	SD CASSI	34.24 on ICVL, 32.71 on Harvard	Unrolled network	Learned network prior
HCS^2^-Net^31^	SD/SS CASSI	34.52 on ICVL (10 scenes), 39.22 on CAVE (SS-CASSI), 29.33 on CAVE (SD-CASSI)	CNN (untrained)	Residual block, attention module, unsupervised learning, hardware code concatenated to the input measurement, deep image prior
Deep-Tensor^45^	SD CASSI	30.92 on ICVL, Harvard and KAIST (best mean)	CNN (untrained)	Learned tensor decomposition

Evaluation results are collected from each original works

**Fig. 5:**
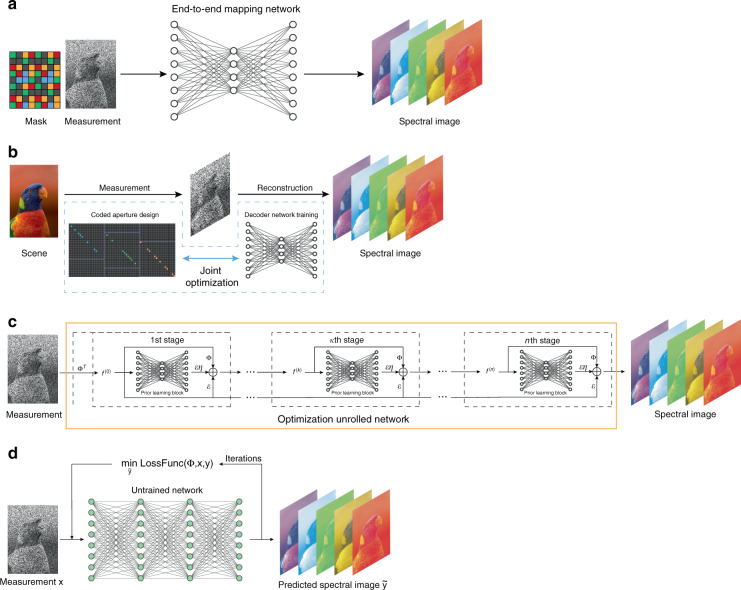
Main ideas of the four deep compressive reconstruction approaches. **a** End-to-end reconstruction. **b** Joint mask learning. **c** Unrolled network. **d** Untrained network

Based on different places deep learning is used, we divide the deep-learning-based compressive reconstruction methods into four categories: (i) *end-to-end reconstruction* that uses deep neural networks for direct reconstruction; (ii) *joint mask learning* that simultaneously learns the coded aperture pattern and the subsequent reconstruction network; (iii) *unrolled network* that unfolds the iterative optimization procedure into a deep network with many stage blocks; (iv) *untrained network* that uses the broad range of the neural network as a prior and performs iterative reconstruction. The main ideas of these four categories are illustrated in Fig. 5.

#### E2E reconstruction

End-to-end (E2E) reconstruction sends measurement into a deep neural network which directly outputs the reconstruction result. Among E2E methods, deep external–internal learning^35^ proposed a novel learning strategy. First, external learning from large dataset was performed to improve the general capability of the network. Then for a specific application, internal learning from single spectral image was used for further improvement. In addition, fusion with panchromatic image showed benefits in improving spatial resolution. *λ*-Net^36^ is an alternative architecture based on conditional generative adversarial network (cGAN). It also adopted self-attention technique and hierarchical reconstruction strategy to promote the performance.

Dataset, network design and loss function are three key factors of the E2E methods. For future improvement, various techniques from RGB patch-wise spectral reconstruction can be employed (see section “RGB Pixel-wise Spectral Reconstruction”). For example, residual blocks, dense structure, and attention module are expected to be adopted. For the choice of loss functions, back-projection pixel loss is suggested to employ, which is beneficial to data fidelity. It simulates the measurement using the known coded aperture pattern and reconstructed spectral image, and compares the simulated back-projected measurement with the ground truth. Novel losses such as feature and style loss can also be attempted.

#### Joint mask learning

Coded aperture relates to sensing matrix Φ involved in spectral image acquisition process. Conventional methods based on CASSI often adopt random coded apertures since the random code can preserve the properties needed for reconstruction (e.g., restricted isometry property, RIP^37^) in high probability. As demonstrated in ref. ^38^, there are approaches for optimizing coded apertures by considering RIP as the criteria. However, such optimization does not present a significant improvement compared to the random coded masks.

In deep compressive reconstruction architecture, coded aperture is seen as an encoder to embed the spectral signatures. Therefore, it should be optimized together with the decoder, i.e., the reconstruction network. HyperReconNet^39^ jointly learns the coded aperture and the corresponding CNN for reconstruction. Coded aperture was appended into the network as a layer, and BinaryConnect method^40^ was adopted to map float digits to binary coded aperture entities. However, most works that used deep learning did not carefully optimize the coded aperture, hence this direction remains to be researched deeper.

#### Unrolled network

Unrolled network unfolds the iterative optimization-based reconstruction procedure into a neural network. In detail, a block of the unrolled network learns the solution of one iteration in the optimization algorithm.

Wang et al.^24^ proposed a hyperspectral image prior network that is adapted from the iterative reconstruction problem. Based on half quadratic splitting (HQS)^41^, they obtained an iterative optimization formula. By using network layers to learn the solution, they unfolded the *K*-iteration reconstruction procedure into a *K*-stage neural network. As a later work, Deep Non-local Unrolling (DNU)^42^ further simplified the formula derived in ref. ^24^ and rearranged the sequential structure in ref. ^24^ into a parallel one. Sogabe et al. proposed an ADMM-inspired network for compressive spectral imaging^43^. They unrolled the adaptive ADMM process into a multi-staged neural network and showed a performance improvement compared to HQS-inspired method^24^.

Unrolled network can boost the reconstruction speed by freezing the parameters of iteration into neural network layers. Each stage has the mission to solve an iteration equation, which makes the neural network explainable.

#### Untrained network

Deep image prior, as proposed in ref. ^44^, states that the structure of a generative network is sufficient to capture image priors for reconstruction. To be more specific, the range of deep neural networks can be large enough to include all common spectral image that we are going to recover. Therefore, carefully-designed untrained network is capable of performing spectral image reconstruction. Though it takes time for the iterative gradient descent procedure, such approach is free from pre-training and has high generalization ability.

Those labeled *untrained* in Table 1 adopted untrained network for compressive spectral reconstruction. The HCS^2^-Net^31^ took random code of the coded aperture and snapshot measurement as the network input, and used unsupervised network learning for spectral reconstruction. They adopted many deep-learning techniques such as residual block and attention module to enhance the network capability. In ref. ^45^, spectral data cube was considered as a 3D tensor and tensor Tucker decomposition^46^ was performed in a learned way. They designed network layers based on Tucker decomposition and used low rank prior of the core tensor, which may be beneficial to better capture the spectral data structure.

## Phase-coded spectral imaging

Phase-coded spectral imaging formulates the image generation as a convolution process between wavelength specified point spread function (PSF) and monochrome object image at each wavelength. The phase encoding manipulates the phase term of the PSF which will distinguish spectral signature as light propagates. Compared with amplitude-coded spectral imaging, phase-coded approach can greatly increase the light throughput (hence the signal-to-noise ratio). Since the phase encoding is mainly operated on a thin DOE, which is easy to attach onto a camera, the phase-coded spectral imaging system can be very compact.

One can recover the spectral signature by designing algorithms with the corresponding DOE (also called *diffuser* in some works^16,47–49^). With the aid of deep learning, these methods displayed comparable performance. Furthermore, benefitting from the depth dependence of diffraction model, they can also obtain depth information apart from spectral signature of a scene^50^.

Phase-coded approach for spectral imaging consists of two parts: (i) phase encoding strategy, often related to the design of DOE; (ii) reconstruction algorithm establishment. In this section, we first describe the phase encoding diffraction model, then introduce deep-learning-empowered works using different phase encoding strategies and systems.

### Diffraction model

**Fig. 6:**
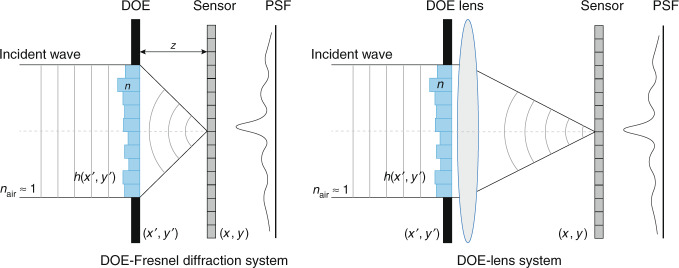
Schematic diagram of diffractive spectral imaging via a diffractive optical element (DOE). The left is the system using a transmissive DOE and a sensor, where the incident wave passes a DOE and then propagates a distance *z* before hitting the sensor. The propagation can be modeled by Fresnel diffraction. The right system uses an imaging lens just behind the DOE. After passing the DOE, the incident wave converged on the sensor through the lens. DOE has a height profile that introduces the phase shift

The phase-coded spectral imaging system is based on previous works of diffractive imaging^51,52^. The system often consists of a DOE (transmissive or reflective) and a bare camera sensor, separated by a distance *z*. As illustrated in Fig. 6, there are two kinds of phase-coded spectral imaging systems, namely DOE-Fresnel diffraction (left) and DOE-Lens system (right), different from whether there is a lens.

#### PSF construction

We use the transmissive DOE for model derivation. PSF *p*_*λ*_(*x*, *y*) is the system response to a point source at the image plane. Suppose the incident wave field at position $(x^{\prime} ,y^{\prime} )$ of the DOE coordinate at wavelength *λ* is

8 $${u}_{0\lambda }(x^{\prime} ,y^{\prime} )\,=\,{A}_{\lambda }(x^{\prime} ,y^{\prime} ){e}^{i{\phi }_{0\lambda }(x^{\prime} ,y^{\prime} )}$$

The wave field first experiences a phase shift *ϕ*_*h*_ determined by the height profile of the DOE:

9 $$\begin{array}{lll}{u}_{1\lambda }(x^{\prime} ,y^{\prime} )&=&{A}_{\lambda }(x^{\prime} ,y^{\prime} ){e}^{i\left[{\phi }_{0\lambda }(x^{\prime} ,y^{\prime} )\,+\,{\phi }_{h}(x^{\prime} ,y^{\prime} )\right]},\\ {\phi }_{h}(x^{\prime} ,y^{\prime} )&=&k{{\Delta }}{n}_{\lambda }h(x^{\prime} ,y^{\prime} )\end{array}$$

where Δ*n* is the refractive index difference between DOE (*n*(*λ*)) and air, *k* = 2*π*/*λ* is the wave number.

For the DOE-lens system, the PSF is^16^:

10 $${p}_{\lambda }(x,y)\,=\,\left|{{{{\mathcal{F}}}}}^{-1}\left[{u}_{1\lambda }(x^{\prime} ,y^{\prime} )\right]\right|$$

where ${{{{\mathcal{F}}}}}^{-1}$ is the inverse 2D Fourier transform due to the Fourier characteristics of the lens.

For DOE-Fresnel diffraction system, the wave field propagates a distance *z* that can be modeled by the Fresnel diffraction law such that *λ* ≪ *z*:

11 $$\begin{array}{lll}{u}_{2\lambda }(x,y)&=&\frac{{e}^{ikz}}{i\lambda z}\iint {u}_{1\lambda }(x^{\prime} ,y^{\prime} ){e}^{\frac{ik}{2z}\left[{(x\,-\,x^{\prime} )}^{2}\,+\,{(y\,-\,y^{\prime} )}^{2}\right]}\,\,{{\mbox{d}}}x^{\prime} {{\mbox{d}}}\,y^{\prime} \\ &=&\frac{{e}^{ikz}}{i\lambda z}\iint {A}_{\lambda }(x^{\prime} ,y^{\prime} ){e}^{i\left[{\phi }_{0\lambda }(x^{\prime} ,y^{\prime} )\,+\,{\phi }_{h}(x^{\prime} ,y^{\prime} )\right]}{e}^{\frac{ik}{2z}\left[{(x\,-\,x^{\prime} )}^{2}\,+\,{(y\,-\,y^{\prime} )}^{2}\right]}\,\,{{\mbox{d}}}x^{\prime} {{\mbox{d}}}\,y^{\prime} \end{array}$$

Finally, for computation convenience, we expand the Eq. (11) and represent it with a Fourier transform ${{{\mathcal{F}}}}$. The final PSF is formulated as

12 $${p}_{\lambda }(x,y)\,\propto\, {\left|{{{\mathcal{F}}}}\left[{A}_{\lambda }(x^{\prime} ,y^{\prime} ){e}^{i\left[{\phi }_{0\lambda }(x^{\prime} ,y^{\prime} )\,+\,{\phi }_{h}(x^{\prime} ,y^{\prime} )\right]}{e}^{i\frac{\pi }{\lambda z}(x{^{\prime} }^{2}\,+\,y{^{\prime} }^{2})}\right]\right|}^{2}$$

#### Image formation

Considering an incident object distribution ${o}_{\lambda }(x^{\prime} ,y^{\prime} )$ at DOE, we can decompose it into integral of object points:

13 $${o}_{\lambda }(x^{\prime} ,y^{\prime} )\,=\,\iint {o}_{\lambda }(\xi ,\eta )\,\cdot\, \delta (x^{\prime} \,-\,\xi ,y^{\prime} \,-\,\eta )\,\,{{\mbox{d}}}\xi {{\mbox{d}}}\,\eta$$

Before hitting the sensor, the spectral distribution is

14 $$\begin{array}{lll}{I}_{\lambda }(x,y)&=&\iint {o}_{\lambda }(\xi ,\eta )\cdot {{{\rm{PSF}}}}\{\delta (x^{\prime} \,-\,\xi ,y^{\prime} \,-\,\eta )\}\,\,{{\mbox{d}}}\xi {{\mbox{d}}}\,\eta \\ &=&\iint {o}_{\lambda }(\xi ,\eta )\,\cdot\, {p}_{\lambda }(x\,-\,\xi ,y-\eta )\,\,{{\mbox{d}}}\xi {{\mbox{d}}}\,\eta \\ &=&{o}_{\lambda }(x,y)\,*\, {p}_{\lambda }(x,y)\end{array}$$

where PSF denotes system response to a point source and *p*_*λ*_ is shifted by *ξ* and *η* in *x* and *y* axis because of the same shift at the point source.

Finally, on the sensor plane (with sensor spectral response *D*), the intensity is

15 $$I(x,y)\,=\,\int _{{{\Lambda }}}\,D(\lambda )\,\cdot\, \left[{o}_{\lambda }(x,y)\,*\, {p}_{\lambda }(x,y)\,\,{{\mbox{d}}}\,\lambda \right]$$

Similar to Fig. 4, vectorize *o*_*λ*_ to **x** and matrixize the convolution with PSF function to Φ, we can discretize Eq. (15) and form the reconstruction problem as Eq. (7). Researchers can use similar optimization algorithms or deep-learning tools for DOE design and spectral image recovery.

### Phase encoding strategies

A good PSF design contributes to the effective phase encoding, which can bring more precise spectral reconstruction results. Based on the slight difference of the imaging system, we categorize the phase encoding strategies below.

#### DOE with Fresnel diffraction

Many phase-coded spectral imaging methods are developed from diffractive computational color imaging. Peng et al.^53^ proposed an optimization-based DOE design approach to obtain a shape invariant PSF towards wavelength. Together with the deconvolution method, they reconstructed high-fidelity color image.

Although the shape invariant PSF^53^ is beneficial for high-quality achromatic imaging, the overlap of PSF at each wavelength causes difficulty on spectral reconstruction, which hinders its application on spectral imaging. Jeon et al.^15^ designed a spectrally varying PSF that regularly rotates with wavelength, which encoded the spectral information. Their rotational PSF design makes it distinct at different wavelength, which is quite suitable for spectral imaging. By putting the resultant intensity image into an optimization-based unrolled network, they achieved high peak signal-to-noise ratio (PSNR) and spectral accuracy in visible wavelength range, within a very compact system.

#### DOE/diffuser with lens

A similar architecture is using DOE (or, diffuser) with an imaging lens closely behind, which is shown in Fig. 6 (right). In 2016, Golub et al.^49^ proposed a simple diffuser-lens optical system and used compressed-sensing-based algorithm for spectral reconstruction. Hauser et al.^16^ extended the work to 2D binary diffuser (for binary phase encoding) and employed a deep neural network (named DD-Net) for spectral reconstruction. They reported high-quality reconstruction in both simulation and lab experiments.

#### Combination with other encoding approach

Combining phase encoding with other encoding architectures is also a feasible approach, and deep learning can handle such complicated combined-architecture model. For example, compressive diffraction spectral imaging method combined DOE for phase encoding with coded apertures for further amplitude encoding^54^. However, the reconstruction progress is very tough, and the light efficiency is not high. Another example is the combination with optical filter array. Based on previous works of lensless imaging^47,55^, Monakhova et al. proposed a spectral DiffuserCam^48^, using a diffuser to spread the point source and a tiled filter array for further wavelength encoding. As the method has a similar mathematical spectral formation model, it is promising to apply deep learning to spectral DiffuserCam’s complex reconstruction task.

## Wavelength-coded spectral imaging

Wavelength-coded spectral imaging uses optical filters to encode spectral signature along wavelength axis. Among wavelength-coded methods, RGB image, which is encoded by RGB narrowband filters, is mostly used. It is necessary to reconstruct the spectral image from the RGB one, because RGB image is commonly used by people, and the corresponding spectral image is fundamental to rendering scenes on monitors. Over the years, researchers have been pursuing fast and accurate approaches of wavelength-coded spectral imaging. They found RGB filters may be suboptimal, thus different narrowband filters as well as self-designed broadband filters are explored.

### Image formation model

We first introduce the image formation model in wavelength encoding context. Consider an intensity *I*_*k*_(*x*, *y*) from a pixel at (*x*, *y*), *k* is the channel index indicating different wavelength modulation. For RGB image, *k* ∈ {1, 2, 3}, representing red, green, and blue. The encoded intensity is generated by the scene reflectance spectra *S* under illumination *E*:

16 $${I}_{k}(x,y)\,=\,\int _{{{\Lambda }}}E(\lambda )S(x,y,\lambda ){Q}_{k}(\lambda )D(\lambda )\,\,{{\mbox{d}}}\,\lambda$$

where *Q*_*k*_ is the *k*th filter transmittance curve, *D* is the camera sensitivity, and Λ is the wavelength range. Illumination distribution *E* and scene spectral reflectance *S* can be combined as the scene spectral radiance *R*:

17 $${I}_{k}(x,y)\,=\,\int _{{{\Lambda }}}R(x,y,\lambda ){Q}_{k}(\lambda )D(\lambda )\,\,{{\mbox{d}}}\,\lambda$$

**Fig. 7:**
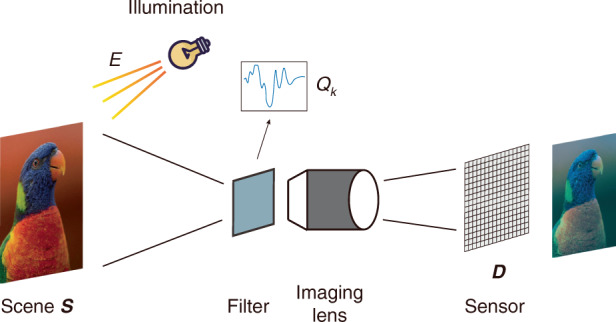
Illustration of wavelength encoding spectral imaging process. The scene *S* is illuminated by the light source *E*, and is wavelength-coded through filters *Q*. Then the encoded scene spectral radiance is captured by the imaging lens on a sensor with spectral response *D*

The imaging process is illustrated in Fig. 7. In practice, we have the encoded object intensities *I* and filter curves *Q*, but the camera sensitivity is sometimes inconvenient to measure, thus many methods assume it be ideally flat. Under experimental conditions, we also know illumination *E*. Then Eq. (17) (or Eq. (16)) becomes an (underdetermined) matrix inversion problem after discretization.

### RGB pixel-wise spectral reconstruction

Early works of wavelength-coded spectral reconstruction is pixel-wise on RGB images. They consider the reduced problem of how to reconstruct a spectrum vector that has more channels from a 3-channel RGB vector, without knowing the camera’s RGB-filter response. In general, these pixel-wise approaches seek a representation of the single spectrum (either manifold embedding or basis functions) and develops methods to reconstruct spectrum from that representation.

There are two modalities of methods on spectrum representation: (i) *spectrum manifold learning* that seeks the hidden manifold embedding space to express the spectrum effectively; (ii) *basis function fitting* that expands the spectrum as a set of basis functions, and fit a small number of coefficients.

#### Spectrum manifold learning

This approach assumes that a spectrum **y** is controlled by a vector **x** in the low-dimensional manifold ${{{\mathcal{M}}}}$ and tries to find the mapping *f* that relates **y** with **x**:

18 $${{{\bf{y}}}}\,=\,f({{{\bf{x}}}}),\quad {{{\bf{y}}}}\,\in\, {{{\mathcal{D}}}},f\,\in\, {{{\mathcal{F}}}},{{{\bf{x}}}}\,\in\, {{{\mathcal{M}}}}$$

where ${{{\mathcal{D}}}}$ is the high-dimensional data space (commonly, ${{{\mathcal{M}}}}\,=\,{{\mathbb{R}}}^{m},{{{\mathcal{D}}}}\,=\,{{\mathbb{R}}}^{n}.m,n\,\in\, {\mathbb{N}}$ is the space dimension). ${{{\mathcal{F}}}}$ is a functional space that contains functions mapping data from ${{{\mathcal{M}}}}$ to ${{{\mathcal{D}}}}$.

Manifold learning assumes a low-dimensional manifold ${{{\mathcal{M}}}}$ embedded in the high-dimensional data space ${{{\mathcal{D}}}}$, and attempts to recover ${{{\mathcal{M}}}}$ from the data drawn in ${{{\mathcal{D}}}}$. Reference^56^ proposed a three-step method: (i) Find an appropriate dimension of the manifold space through Isometric Feature Mapping (Isomap^57^); (ii) Train a radial basis function (RBF) network to embed the RGB vector in ${{{\mathcal{M}}}}$, which determines the inverse of *f* in Eq. (18); (iii) Use dictionary learning to map the manifold representation in ${{{\mathcal{M}}}}$ back to the spectra space, which determines the function *f* in Eq. (18). The RBF network and dictionary learning method can be substituted by deep neural networks (such as AutoEncoder) to improve the performance, hence the manifold-based reconstruction can be further promoted.

#### Basis function fitting

This approach assumes that a spectrum **y** = *y*(*λ*) is expanded by a set of basis functions {*ϕ*_1_(*λ*), … , *ϕ*_*N*_(*λ*)}:

19 $$y(\lambda )\,=\,\mathop{\sum }\limits_{i\,=\,1}^{N}{\alpha }_{i}{\phi }_{i}(\lambda )$$

where *α* are the coefficients to fit.

In a short note by Glassner^17^, a simple matrix inversion method was developed for RGB-to-spectrum conversion, but the resultant spectrum only has three nonzero components, which is rare in real world. At the end of the note, the author reported a weighted basis function fitting approach to construct spectrum from RGB triplet, with constant, sine, and cosine three functions. To render light interference, Sun et al.^18^ compared different basis functions for deriving spectra from colors and proposed an adaptive method that uses Gaussian functions. Nguyen et al.^20^ further developed the basis function approach, proposing a data-driven method that learns RBF to map illumination normalized RGB image to spectral image.

In ref. ^21^, an over-complete hyperspectral dictionary was constructed using K-SVD algorithm from the proposed dataset, which contained a set of nearly orthogonal vectors that can be seen as learned basis functions. Similar to the dictionary learning approach, deep-learning tools can be used for learning basis functions. In ref. ^58^, basis functions are generated during training, and coefficients are predicted through a U-Net at test time. It is very computationally efficient since it only needs to fit a small number of coefficients during the test time. Although the spectral reconstruction accuracy is not as high as other CNN-based methods (which sufficiently extract spectral patch correlation), it is the fastest method in NTIRE 2020 with reconstruction time only 34 ms per image.

### RGB Patch-wise spectral reconstruction

As reported in ref. ^22^, spectra within an image patch has certain correlation. However, pixel-wise approaches cannot exploit such correlation, which may lead to poor reconstruction accuracy in comparison with patch-wise approaches. In ref. ^59^, a handmade patch feature through convolution operation was proposed, which extracts neighborhood feature of a RGB pixel from the training spectral dataset. This work gave a practical idea of how to utilize such patch feature in a spectral image, which is just suitable for convolutional neural networks (CNNs).

CNNs can perform more complex feature extraction through multiple convolution operators. In 2017, Xiong et al. proposed HSCNN^23^ to apply a CNN on up-sampled RGB and amplitude-coded measurements for spectral reconstruction. At the same year, Galliani et al. proposed learned spectral super-resolution^60^, using a CNN for end-to-end RGB to spectral image reconstruction. Their works obtained good spectral reconstruction accuracy on many open spectral datasets, encouraging later works on CNN-based spectral reconstruction. The number of similar works grew rapidly as New Trends in Image Restoration and Enhancement (NTIRE) challenge was hosted in 2018^61^ and 2020^25^, where many deep-learning groups joined in and contributed to the exploitation of various network structures for spectral reconstruction.

Neural network-based methods takes the advantage of deep learning and can better grasp the patch spectra correlation. Diverse network structures as well as advanced deep-learning techniques are exploited by different works, which are arranged in Table 2.

**Table 2:** Comparison of neural network-based works for end-to-end spectral reconstruction from RGB images

Method	Network Backbone	Deep-Learning Techniques	Evaluation (RMSE)	Evaluation (MRAE)
HSCNN^23^	CNN	Multi-layer CNN	17.006 on Clean track (NTIRE 2018)	0.0190 on Clean track (NTIRE 2018)
Adv_rgb2hs^62^	cGAN	Additional MAE loss on cGAN	1.457 on ICVL, 24.81 on Clean, 34.05 on Real World (NTIRE 2018)	0.0218 on Clean, 0.0396 on Real World (NTIRE 2018)
Spectral super-resolution^60^	Densenet	Dense structure, sub-pixel convolution layer	1.98 on ICVL, 5.27 on NUS, 4.76 on CAVE	/
HSCNN+^71^	2-level CNN	Residual blocks, dense structure, feature fusion^70^	13.128[14.45] on Clean, 22.935[24.06] on Real World (NTIRE 2018)	0.0135[0.0137] on Clean, 0.0293[0.0310] on Real World (NTIRE 2018)
LFB^98^	U-Net	Camera sensitivity prior	20.146[16.19] on Clean, 27.557[26.44] on Real World (NTIRE 2018)	0.01704 on Clean, 0.03081 on Real World (NTIRE 2018)
CVL^99^	2-level CNN	Residual blocks, PReLU activation	1.23 on ICVL, 3.66 on NUS, 3.5275 on CAVE, 17.27 on Clean, 27.09 on Real World (NTIRE 2018)	0.0174[0.0152] on Clean, 0.0364[0.0335] on Real World (NTIRE 2018)
3D-CNN^100^	CNN	3D CNN^101^	1.115 on ICVL, 2.86 on CAVE, 20.010[19.41] on Clean (NTIRE 2018)	0.018[0.0181] on Clean track (NTIRE 2018)
Sensitivity estimation^79^	CNN	Back-projection loss	(s) 0.0282 on ICVL, 0.0316 on CAVE	(s) 0.13 on ICVL 0.38 on CAVE
Deep Function-Mixture Network^68^	multi-level CNN	Pixel attention, feature fusion	4.54 on CAVE, 2.54 on Harvard, 1.03 on NTIRE 2018, 0.01268 on Clean, 0.01946 on Real World (NTIRE 2020)	0.03075 on Clean, 0.06212 on Real World (NTIRE 2020)
MXR-U-Nets^102^	U-Net	XResnet block^103^, Mish activation function^104^, feature and style loss, self-attention layer	0.01645 on Clean, 0.02255 on Real World (NTIRE 2020)	0.0454[0.04441] on Clean, 0.0840[0.09322] on Real World (NTIRE 2020)
AWAN^80^	CNN	Residual blocks, channel attention, PReLU activation, back-projection loss, self ensemble, model ensemble	0.0111[0.01293] on Clean, 0.0170[0.01991] on Real World (NTIRE 2020); 10.24 on Clean, 21.33 on Real World (NTIRE 2018)	0.0312[0.03010] on Clean, 0.0639[0.06210] on Real World (NTIRE 2020), 0.0114 on Clean, 0.0277 on Real World (NTIRE 2018)
RPAN^72^	CNN	Pixel attention, global residual connection, feature fusion	4.301[0.01695] on Clean, 4.984[0.02071] on Real World (NTIRE 2020)	0.03756[0.03601] on Clean, 0.06787[0.06780] on Real World (NTIRE 2020)
HRNet^73^	4-level CNN	Residual blocks, dense structure, feature fusion, sub-pixel convolution layer, model ensemble	0.01354[0.01389] on Clean, 0.01786[0.01923] on Real World (NTIRE 2020)	0.04233[0.03231] on Clean, 0.06825[0.06200] on Real World (NTIRE 2020)
C2H-Net^92^	U-Net	Additional category prior	4.7313 on CAVE	/
Double Ghost^105^	GhostNet	GhostNet block^106^, residual connection, attention mechanism, non-local block^107^, PReLU activation	0.0162 on Real World track (NTIRE 2020)	0.0439 on Real World track (NTIRE 2020)

In the network architecture column, *level* means parallel CNN layers for data flow. For the *deep-learning techniques* column, we highlight the techniques that may play an important role in the method’s performance. Performance evaluations are collected from reported results of the original article, corresponding articles or NTIRE competition. Evaluation results from the original article is considered first, then NTIRE competition, and finally the corresponding articles. If the evaluation results occurred in both the original article and the NTIRE competition report, we use [] to denote the evaluation result in the NTIRE report. Evaluation values labeled “s” in the table are from the scaled dataset (datasets that are linearly scaled to [0,1] range)

#### Leveraging advanced deep-learning techniques

We can gain some inspirations from Table 2. First, most works are CNN-based, this perhaps because CNN can better extract patch spectral information than generative adversarial networks (GANs). There was a work based on conditional GAN (cGAN)^62^, which takes RGB image as conditional input. They also used *L*_1_ distance loss (mean absolute error loss) as ref. ^63^ to encourage less blur, but the reconstruction accuracy was not better than HSCNN^23^ (ref. ^62^ has relative root-mean-square error (RMSE) 0.0401 on ICVL dataset, while HSCNN has 0.0388).

Moreover, many advanced deep-learning techniques are introduced and shown to be effective. For instance, residual blocks^64^ and dense structure^65^ become increasingly common. This is because residual connection can broaden the network’s receptive field and dense structure can enhance the feature passing process, resulting in better extraction of spectral patch correlation. Attention mechanism^66^ is a popular deep-learning technique and is also introduced in spectral imaging works. For spectral reconstruction, there are two kinds of attention: spatial attention (e.g., the self-attention layer^67,68^) and spectral attention (channel attention^69^). Attention module learns a spatial or spectral weight, helping the network focus on the informative parts of the spectral image. Feature fusion is the concatenation of multiple parallel layers, which was researched in ref. ^70^. It was adopted in refs. ^71–73^ and showed positive influence on spectral reconstruction. Finally, ensemble technique is encouraged to further promote the network performance. Model ensemble and self ensemble are two kinds of ensemble strategies. Model ensemble averages networks that are retrained with different parameters, while self ensemble averages the results of transformed input to the same network. Single network may fall into local minimum, which leads to poor generalization performance. By applying the ensemble technique, one can fuse the knowledge of multiple networks or different viewpoint to the same input. HRNet^73^ adopted model ensemble, and it showed improvement on reconstruction result.

Since the spectral reconstruction is a kind of image-to-image task, many works borrow effective deep-learning techniques from other image-to-image tasks, such as U-Net architecture from^74^ segmentation task, sub-pixel convolution layer^75^, channel attention^69^ from image super resolution task, and feature loss and style loss from image style transfer task^76,77^. This is also a way to introduce advanced deep-learning techniques into spectral reconstruction.

#### Towards illumination invariance

Object reflectance spectrum without illumination is a desired objection for spectral reconstruction, since it honestly reflects the scene components and properties. To recover object reflectance, one need to strip out environment illumination *E* from scene spectral radiance *R*, but it is inconvenient to measure the illumination spectra. Researchers often use illumination invariant property of the object spectrum to remove attached illumination from the scene radiance.

Reference^20^ proposed an approach to employ illumination invariance. They proposed RGB white-balancing to normalize the scene illumination. As an additional product, they can estimate the environment illumination by comparing reconstructed scene with the original scene. In ref. ^78^, Denoising AutoEncoder (DAE) was used to obtain robust spectrum from noised input, which contains original spectrum under different illumination conditions. Through this many-to-one mapping, reconstruction to spectrum became invariant to illumination.

#### Utilizing RGB-filter response

RGB-filter response is the wavelength encoding function *Q* in Eq. (16). In many works^79,80^, the RGB-filter response is termed *camera spectral sensitivity* (CSS) prior. To avoid semantic ambiguity of CSS and camera response *D* in Eq. (16), we substitute it with *RGB-filter response*.

RGB-filter response is not always accessible for practical applications, which is a notable problem. A common way to tackle it is using CIE color mapping function for simulation^81^. Reference^79^ proposed another solution to address this problem. They adopted a classification neural network to estimate a suitable RGB-filter response from the given camera sensitivity set. Then they can use the estimated filter response function and another network to recover the spectral signature. These two nets were trained together via a united loss function.

When RGB-filter response is known, RGB image can be reconstructed from spectral image, thus back-projection (or perceptual) loss can be used. Experiments have shown benefits to add the filter response prior in reconstruction. For example, AWAN^80^, who ranked 1st in NTIRE 2020 Clean track, adopted filter response curves in loss function and got a slight improvement on MRAE metric.

In ref. ^82^, the RGB-filter response *Q* is carefully exploited. They demonstrated that the reconstructed spectrum should follow the color fidelity property *Q*^*T*^*ψ*(*I*) = *I*, where *ψ* is the RGB-to-spectrum mapping and *I* is the RGB pixel intensity.

**Fig. 8:**
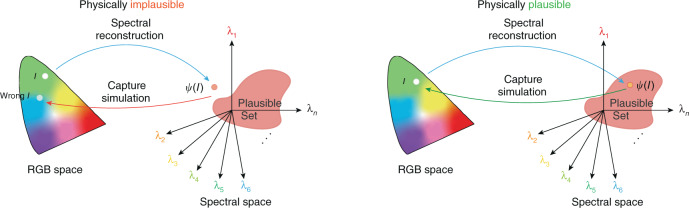
Illustration of the physically implausible and plausible set. Physically implausible set (left) is the spectra points that cannot be mapped to the original RGB point by the RGB-filter response, while physically plausible set (right) is those spectra points that can be mapped back

They defined the set of spectra that satisfy color fidelity as *plausible set*:

$${{{\mathcal{P}}}}(I;Q)\,=\,\left\{{{{\bf{r}}}}| {Q}^{T}{{{\bf{r}}}}\,=\,I\right\}$$

where **r** is spectrum. The concept of *physically plausible* was illustrated in Fig. 8.

They suggest that the reconstructed spectrum should contain two parts: one from the space spanned by three column filter response vectors in *Q*, and the other from the orthogonal complement space of the former. Formally, there exists an orthogonal basis $B\in {{\mathbb{R}}}^{n-3}$ such that *B*^*T*^*Q* = **0**. Therefore, the spectrum to be reconstructed can be expanded as

20 $${{{\bf{r}}}}\,=\,{{{{\rm{P}}}}}^{{{{\rm{Q}}}}}{{{\bf{r}}}}\,+\,{{{{\rm{P}}}}}^{{{{\rm{B}}}}}{{{\bf{r}}}}$$

where P^Q^ and P^B^ are projection operators. Note that *P*^*Q*^ can be precisely calculated in advance, which reduces the reconstruction calculation by 3 dimensions. The remaining task is estimating the spectrum vector in an orthogonal space of filter response vectors, which can be done by training a deep neural network.

### Beyond RGB filters

Since the RGB image has limited information, researchers tend to manually add more information before reconstruction. There are two ways to realize this: (i) using self-designed broadband wavelength encoding to expand the modulation range; (ii) increasing the number of encoding filters. Works in this area mainly use deep-learning tools to design filter response curves and perform spectral reconstruction^83–85^, since the modulation design and the reconstruction process are complicated in computation.

#### Using broadband filters

Based on the idea that traditional RGB camera’s spectral response function is suboptimal for spectrum reconstruction, Nie et al.^83^ employed CNNs to design filter response functions and jointly reconstruct spectral image. They observed the similarity between camera filter array and convolutional layer kernel (the Bayer filter mosaic is similar to a 2 × 2 convolution kernel) and used camera filters as a hardware-implementation layer of the network. Their result showed improvement than traditional RGB-filter-based methods. However, limited by the filter manufacture technology, they only considered filters that were commercially available.

**Fig. 9:**
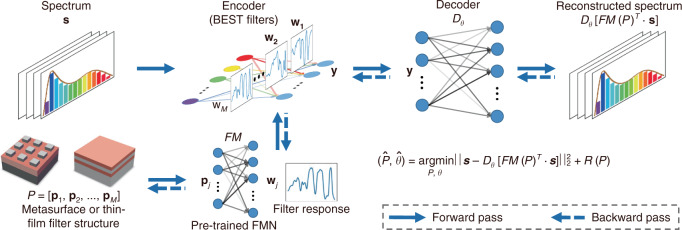
Schematic of the PCSED framework. The broadband encoding stochastic (BEST) filters act as an encoding neural network (encoder), where the learned weights is the filter responses. The weights are constrained by the filters' structure parameters, and are generated from a pre-trained structure-to-response forward modeling network (FMN). The figure is reprinted from ref. ^84^ under a CC BY license (Creative Commons Attribution 4.0 International license)

With the maturity of the modern filter manufacture technology, flexible designed filters with specific response spectrum becomes realizable. Song et al. presented a joint learning framework for broadband filter design, named parameter constrained spectral encoder and decoder (PCSED)^84^, as illustrated in Fig. 9.

They jointly trained filter response curves (as spectral encoder) and decoder network for spectral reconstruction. Benefited from the development of thin-film filter manufacture industry, they can design various filter response functions that are favored by the decoder. They extended the work in ref. ^85^ and got impressive results. The developed hardware, broadband encoding stochastic (BEST) camera, demonstrated great improvements on noise tolerance, reconstruction speed and spectral resolution (301 channels). For the future direction, anti-noise optical filters produced from meta-surface is promising with the development of meta-surface theory and industry^86^.

#### Increasing filter number

Increasing filter number is a straightforward approach to enhance reconstruction accuracy by providing more encoding information. However, this will inevitably lead to bulky system volume. An alternative way to perform wavelength modulation is using liquid crystal (LC). In this way, changing the voltage will switch LC to a different modulation, thus it is convenient to use multiple modulations by applying different voltages. By fast changing the voltage on LC, multiple wavelength encoding operators can be obtained, which is equivalent to increasing filter numbers.

Based on different responses of the LC phase retarder to different wavelengths, the Compressive Sensing Miniature Ultra-Spectral Imager (CS-MUSI) architecture can modulate the spectra like multiple optical filters. Oiknine et al. reviewed spectral reconstruction with CS-MUSI instrument in ref. ^87^. They also proposed DeepCubeNet^88^ that adopted CS-MUSI system to perform 32 different wavelength modulations and used CNN for spectral image reconstruction.

## Spectral imaging datasets

Spectral dataset that contains realistic spectral-RGB image pairs are important for data-driven spectral imaging methods, especially for those using deep learning. CAVE^89^, NUS^20^, ICVL^21^ and KAIST^30^ are the most often used datasets for training and evaluation in spectral reconstruction researches. Other datasets like Harvard^22^, Hyperspectral & Color Imaging^90^, Scyllarus hyperspectral dataset^91^, C2H^92^ are also available. To promote the research of spectral reconstruction from RGB images, competitions were held on 2018 and 2020, where ICVL-expanded dataset (NTIRE 2018^61^) and larger-than-ever database NTIRE 2020^25^ were provided. We summarize the public available spectral image datasets in the following tables. Table 3 gives an overview of the spectral datasets and Table 4 provides a detailed description of the data.

**Table 3:** Spectral dataset parameters

Dataset Name	Year	Spectral range/nm	Channel step/nm	Resolution	Capacity/images
CAVE^89^	2008	400–700	10	512 × 512	32
Harvard^22^	2011	420–720	10	1040 × 1392	75
NUS^20^	2014	400–700	10	1312 × >950	66
ICVL^21^	2016	400–1000, 400–700	about 1.25, 10	1392 × 1300	201
KAIST^30^	2017	420–720	10	2704 × 3376	30
NTIRE 2018^61^	2018	400–700	10	1392 × 1300	256
NTIRE 2020^25^	2020	400–700	10	482 × 512	460
Hyperspectral and color imaging^90^	/	/	/	/	191
Scyllarus^91^	/	400–700	10	1040 × 1392	73
C2H-Data^92^	2020	374.1–988.1, 450–740	about 4.6, 10	1392 × 1650	697

**Table 4:** Description of spectral datasets

Dataset name	Description
CAVE^89^	Contains diverse objects and materials; taken under CIE Standard Illuminant D65 illumination.
Harvard^22^	Contains indoor and outdoor scenes; 50 were taken under daylight illumination, 25 under artificial and mixed illumination.
NUS^20^	Contains outdoor images of natural objects, man-made objects, buildings; taken under artificial wideband lights of different color temperatures, illumination spectra is provided.
ICVL^21^	Contains urban, suburban, rural, indoor and plant-life scenes; taken under natural light.
KAIST^30^	Contains color checkerboards with objects for network evaluation; use Xenon Illumination (Thorlab HPLS-30-4) as the light source.
Hyperspectral and color imaging^90^	Contains 88 outdoor scenes, 57 fruits, 46 color charts and patches; taken under different lighting conditions, illumination data was provided.
Scyllarus^91^	Contains portraits, office scenes, close-ups, fruits and flowers, landscapes, each has around 15 images; office scenes were taken under fluorescent lighting, others under natural lighting.
C2H-Data^92^	Contains various real and artificial fruits and vegetables; taken under Tungsten-bromine lamps.

Two problems still exist for these datasets: (i) insufficient capacity for extracting high-complexity spatial–spectral feature; (ii) unfixed train-test split. Some datasets don’t provide a fixed train-test split, causing unfair comparison among methods that use different train-test split strategy. Therefore, it is important to have a large but standard database. We hope the database has unparalleled scale, accuracy and diversity to boost future researches.

At present phase when such a giant standard dataset is not available, we think the popular datasets ICVL^21^, CAVE^89^, NUS^20^ and KAIST^30^ are sufficient for the reconstruction accuracy analysis on both spatial and spectral domain.

### Spectral image quality metrics

There are numerous metrics used for performance evaluation in spectral reconstruction, and we refer to ref. ^93^ for their definition and comparison.

In general, PSNR, structural similarity (SSIM) index and spectral angle map (SAM) are mostly used for amplitude-coded methods, while different metrics like root-mean square error (RMSE) and mean relative absolute error (MRAE) are applied on wavelength-coded methods. As a consequence, it is inconvenient to compare the performance between wavelength-coded and amplitude-coded methods. Therefore, for the convenience of the community to compare different methods, it is necessary to set unified metrics. We think some common metrics are needed for the comparison between the two methods. For example, SSIM, RMSE and RMAE can be employed by both methods at evaluation.

Furthermore, we also need metrics to compare the reconstruction speed. Different works perform spectral reconstruction for different resolution images on various computing devices. We think *pixel reconstruction speed* is a reliable metric to compare reconstruction speed. It is the average speed on test dataset divided by the the 3D resolution of the data (i.e., total pixels of the spectral data used for testing).

## Conclusions and future directions

We have summarized different computational spectral reconstruction methods that adopted deep neural networks, detailing their working principles and deep-learning techniques, under three encoding-decoding modalities: (i) Amplitude-coded. It uses coded aperture for amplitude encoding and is a compressive spectral imaging approach, which exploits compressive sensing theory and iterative optimization process for spectral reconstruction. Based on this feature, some learned reconstruction algorithms are designed to reduce the time consumption for optimization (e.g., unrolled networks), or use deep neural networks to improve the optimization accuracy (e.g., untrained networks). (ii) Phase-coded. It uses DOE to modulate the phase of the input light for each wavelength, and is physically based on Fresnel propagation to expand such phase modulation onto the resultant image. By leveraging creative design of DOE, it enjoys the compactness of the system and improved light throughput. (iii) Wavelength-coded. A common case of wavelength encoding is the RGB image. RGB-to-spectrum is essential in computational graphics, for the benefit of easy-tuning in rendering scenes with spectra on monitors. To extract spectra feature from the RGB data, deep-learning algorithms either map them to a manifold space, or explore the inherent spatial–spectral correlation. As an extension of the RGB-based approaches, multiple self-designed broadband filters for wavelength encoding is developed in recent years. It is more advantageous in the reconstruction precision of the spectra, but the results are also sensitive to the filter fabrication error and imaging noise.

For future directions, extra scene information is expected to promote the reconstruction performance on specific application. In C2H-Net^92^, object category and position was used as a prior, similar to the famous object detection framework YOLO^94^. Based on the observation that pixel patches with object information was often more important than background environment, they introduced object category and position into the reconstruction process. Using additional information can also benefit functional applications of spectral imaging. As a later work of C2H-Net, ref. ^95^ contributes to objection detection using spectral imaging with additional object information.

Additionally, joint encoder-decoder training is also an important direction. Encoder is the hardware layer before the reconstruction algorithm, such as coded aperture, DOE, or optical filter. Simultaneously training the encoder and decoder can provide the decoder with the coding information, thereby improving the performance^39,84^. However, two problems are waiting to be addressed. (i) Finding more efficient encoding hardware and modeling it into a network layer, such as using DOE to improve the light throughput. CS-MUSI architecture that can replace multiple filters^88^ is also encouraged to explore. (ii) Overcoming gradient vanishment. Since the hardware layer is the first layer of the whole deep neural network, when gradient propagates back, it is always very small, which in turn confines the possible change of the hardware layer. If the above two problems are elegantly solved, we believe the deep-learning-empowered computational spectral imaging can step further.

The past decade has witnessed a rapid expansion of deep neural networks in spectral imaging. Despite the success of deep learning, it still has a lot of room for further optimization. Reinforcement learning (RL) is a promising technique to improve the performance. To date, it proves useful to employ RL in finding optimal reconstruction network architectures (i.e., neural architecture search, NAS^96^). With the improvement of computing power, such techniques are promising to increase the performance of learned spectral imaging methods.

Finally, we think transformer-based large-scale deep-learning models have great potential in spectral reconstruction task. Transformer, first applied to the field of natural language processing, is a type of deep neural network mainly based on the self-attention mechanism^66^. It presents strong representation capabilities and has been widely applied in vision tasks^97^. However, such large-scale deep neural networks require huge data for training, hence large-than-ever spectral datasets are demanded, as suggested in section “Spectral Imaging Datasets”.

## References

[1] Shaw GA, Burke HHK (2003). Spectral imaging for remote sensing. Lincoln Lab. J..

[2] Lu GL, Fei BW (2014). Medical hyperspectral imaging: a review. J. Biomed. Opt..

[3] Li QL (2013). Review of spectral imaging technology in biomedical engineering: achievements and challenges. J. Biomed. Opt..

[4] Liang HD (2012). Advances in multispectral and hyperspectral imaging for archaeology and art conservation. Appl. Phys. A.

[5] Feng YZ, Sun DW (2012). Application of hyperspectral imaging in food safety inspection and control: a review. Crit. Rev. Food Sci. Nutr..

[6] Vane G (1993). The airborne visible/infrared imaging spectrometer (AVIRIS). Remote Sens. Environ..

[7] Green RO (1998). Imaging spectroscopy and the airborne visible/infrared imaging spectrometer (AVIRIS). Remote Sens. Environ..

[8] Rickard, L. J. et al. HYDICE: an airborne system for hyperspectral imaging. Proceedings of SPIE 1937, Imaging Spectrometry of the Terrestrial Environment, 173–179 (SPIE, 1993).

[9] Basedow, R. W., Carmer, D. C. & Anderson, M. E. HYDICE system: implementation and performance. Proceedings of SPIE 2480, Imaging Spectrometry, 258–267 (SPIE, 1995).

[10] Gat, N. Imaging spectroscopy using tunable filters: a review. Proceedings of SPIE 4056, Wavelet Applications VII, 50–64 (SPIE, 2000).

[11] Gupta, N. Hyperspectral imager development at army research laboratory. Proceedings of SPIE 6940, Infrared Technology and Applications XXXIV, 69401P (SPIE, 2008).

[12] Hagen NA, Kudenov MW (2013). Review of snapshot spectral imaging technologies. Opt. Eng..

[13] Candès EJ, Romberg J, Tao T (2006). Robust uncertainty principles: exact signal reconstruction from highly incomplete frequency information. IEEE Trans. Inform. Theory.

[14] Donoho DL (2006). Compressed sensing. IEEE Trans. Inform. Theory.

[15] Jeon DS (2019). Compact snapshot hyperspectral imaging with diffracted rotation. ACM Trans. Graph..

[16] Hauser J (2020). DD-Net: spectral imaging from a monochromatic dispersed and diffused snapshot. Appl. Opt..

[17] Glassner AS (1989). How to derive a spectrum from an RGB triplet. IEEE Computer Graph. Appl..

[18] Sun YL (1999). Deriving spectra from colors and rendering light interference. IEEE Computer Graph. Appl..

[19] Smits B (1999). An RGB-to-spectrum conversion for reflectances. J. Graph. Tools.

[20] Nguyen, R. M. H., Prasad, D. K. & Brown, M. S. Training-based spectral reconstruction from a single RGB image. Proceedings of the 13th European Conference on Computer Vision, 186–201 (Springer, 2014).

[21] Arad, B. & Ben-Shahar, O. Sparse recovery of hyperspectral signal from natural RGB images. Proceedings of the 14th European Conference on Computer Vision, 19–34 (Springer, 2016).

[22] Chakrabarti, A. & Zickler, T. Statistics of real-world hyperspectral images. Proceedings of CVPR 2011. Colorado Springs: IEEEE, 2011, 193–200.

[23] Xiong, Z. W. et al. HSCNN: CNN-based hyperspectral image recovery from spectrally undersampled projections. Proceedings of 2017 IEEE International Conference on Computer Vision Workshops, 518–525 (IEEE, 2017).

[24] Wang, L. Z. et al. Hyperspectral image reconstruction using a deep spatial-spectral prior. Proceedings of 2019 IEEE/CVF Conference on Computer Vision and Pattern Recognition, 8024–8033 (IEEE, 2019).

[25] Arad, B. et al. NTIRE 2020 challenge on spectral reconstruction from an RGB image. Proceedings of 2020 IEEE/CVF Conference on Computer Vision and Pattern Recognition Workshops, 1806–1822 (IEEE, 2020).

[26] Gehm ME (2007). Single-shot compressive spectral imaging with a dual-disperser architecture. Opt. Express.

[27] Wagadarikar A (2008). Single disperser design for coded aperture snapshot spectral imaging. Appl. Opt..

[28] Correa CV, Arguello H, Arce GR (2015). Snapshot colored compressive spectral imager. J. Opt. Soc. Am. A.

[29] Lin X (2014). Spatial-spectral encoded compressive hyperspectral imaging. ACM Trans. Graph..

[30] Choi I (2017). High-quality hyperspectral reconstruction using a spectral prior. ACM Trans. Graph..

[31] Sun YB (2022). Unsupervised spatial-spectral network learning for hyperspectral compressive snapshot reconstruction. IEEE Trans. Geosci. Remote Sens..

[32] Rueda, H., Arguello, H. & Arce, G. R. Compressive spectral imaging based on colored coded apertures. Proceedings of 2014 IEEE International Conference on Acoustics, Speech and Signal Processing, 7799–7803 (IEEE, 2014).

[33] Yuan, X. Generalized alternating projection based total variation minimization for compressive sensing. Proceedings of 2016 IEEE International Conference on Image Processing, 2539–2543 (IEEE, 2016).

[34] Boyd, S. et al. Distributed optimization and statistical learning via the alternating direction method of multipliers. Found. *Trends Machine Learn*. **3**, 1–122 (2011).

[35] Zhang, T. et al. Hyperspectral image reconstruction using deep external and internal learning. Proceedings of 2019 IEEE/CVF International Conference on Computer Vision, 8558–8567 (IEEE, 2019).

[36] Miao, X. et al. *λ* -Net: reconstruct hyperspectral images from a snapshot measurement. Proceedings of 2019 IEEE/CVF International Conference on Computer Vision, 4058–4068 (IEEE, 2019).

[37] Candès EJ (2008). The restricted isometry property and its implications for compressed sensing. Comptes Rendus Math..

[38] Arce, G. R. et al. Snapshot compressive multispectral cameras. in Wiley Encyclopedia of Electrical and Electronics Engineering (ed. Webster, J. G.) 1–22 (Wiley, 2017).

[39] Wang LZ (2019). HyperReconNet: Joint coded aperture optimization and image reconstruction for compressive hyperspectral imaging. IEEE Trans. Image Proc..

[40] Courbariaux, M., Bengio, Y. & David, J. P. BinaryConnect: training deep neural networks with binary weights during propagations. Proceedings of the 28th International Conference on Neural Information Processing Systems, 3123–3131 (NIPS, 2015).

[41] Geman D, Yang CD (1995). Nonlinear image recovery with half-quadratic regularization. IEEE Trans. Image Proc..

[42] Wang, L. Z. et al. DNU: Deep non-local unrolling for computational spectral imaging. Proceedings of 2020 IEEE/CVF Conference on Computer Vision and Pattern Recognition, 1658–1668 (IEEE, 2020).

[43] Sogabe, Y. et al. Admm-inspired reconstruction network for compressive spectral imaging. Proceedings of 2020 IEEE International Conference on Image Processing, 2865–2869(IEEE, 2020).

[44] Lempitsky, V., Vedaldi, A. & Ulyanov, D. Deep image prior. Proceedings of 2018 IEEE/CVF Conference on Computer Vision and Pattern Recognition, 9446–9454 (IEEE, 2018).

[45] Bacca J, Fonseca Y, Arguello H (2021). Compressive spectral image reconstruction using deep prior and low-rank tensor representation. Appl. Opt..

[46] Kim, Y. D. & Choi, S. Nonnegative tucker decomposition. Proceedings of 2007 IEEE Conference on Computer Vision and Pattern Recognition, 1–8 (IEEE, 2007).

[47] Antipa N (2018). DiffuserCam: lensless single-exposure 3D imaging. Optica.

[48] Monakhova K (2020). Spectral DiffuserCam: lensless snapshot hyperspectral imaging with a spectral filter array. Optica.

[49] Golub MA (2016). Compressed sensing snapshot spectral imaging by a regular digital camera with an added optical diffuser. Appl. Opt..

[50] Baek, S. H. et al. Single-Shot Hyperspectral-Depth Imaging With Learned Diffractive Optics. Proceedings of the IEEE/CVF International Conference on Computer Vision, 2651–2660 (IEEE, 2021).

[51] Peng YF (2015). Computational imaging using lightweight diffractive-refractive optics. Opt. Express.

[52] Heide F (2016). Encoded diffractive optics for full-spectrum computational imaging. Sci. Rep..

[53] Peng YF (2016). The diffractive achromat full spectrum computational imaging with diffractive optics. ACM Trans. Graph..

[54] Kar OF, Oktem FS (2019). Compressive spectral imaging with diffractive lenses. Opt. Lett..

[55] Monakhova K (2019). Learned reconstructions for practical mask-based lensless imaging. Opt. Express.

[56] Jia, Y. et al. From RGB to spectrum for natural scenes via manifold-based mapping. Proceedings of 2017 IEEE International Conference on Computer Vision, 4715–4723 (IEEE, 2017).

[57] Tenenbaum JB, De Silva V, Langford JC (2000). A global geometric framework for nonlinear dimensionality reduction. Science.

[58] Fubara, B. J., Sedky, M. & Dyke, D. RGB to spectral reconstruction via learned basis functions and weights. Proceedings of 2020 IEEE/CVF Conference on Computer Vision and Pattern Recognition Workshops. 1984–1993 (IEEE, 2020).

[59] Robles-Kelly, A. Single image spectral reconstruction for multimedia applications. Proceedings of the 23rd ACM International Conference on Multimedia, 251–260 (ACM, 2015).

[60] Galliani, S. et al. Learned spectral super-resolution. https://arxiv.org/abs/1703.09470 (2017).

[61] Arad, B. et al. NTIRE 2018 challenge on spectral reconstruction from RGB images. Proceedings of 2018 IEEE/CVF Conference on Computer Vision and Pattern Recognition Workshops, 1042–104209 (IEEE, 2018).

[62] Alvarez-Gila, A., Van De Weijer, J. & Garrote, E. Adversarial networks for spatial context-aware spectral image reconstruction from RGB. Proceedings of 2017 IEEE International Conference on Computer Vision Workshops, 480–490 (Venice: IEEE, 2017).

[63] Isola, P. et al. Image-to-image translation with conditional adversarial networks. Proceedings of 2017 IEEE Conference on Computer Vision and Pattern Recognition, 5967–5976 (IEEE, 2017).

[64] He, K. M. et al. Deep residual learning for image recognition. Proceedings of 2016 IEEE Conference on Computer Vision and Pattern Recognition, 770–778 (Las Vegas: IEEE, 2016).

[65] Huang, G. et al. Densely connected convolutional networks. Proceedings of 2017 IEEE Conference on Computer Vision and Pattern Recognition, 2261–2269 (IEEE, 2017).

[66] Vaswani, A. et al. Attention is all you need. Proceedings of the 31st International Conference on Neural Information Processing Systems, 6000–6010 (NIPS, 2017).

[67] Zhang, H. et al. Self-attention generative adversarial networks. Proceedings of the 36th International Conference on Machine Learning, 7354–7363 (PMLR, 2019).

[68] Zhang, L. et al. Pixel-aware deep function-mixture network for spectral super-resolution. Proceedings of the 34th AAAI Conference on Artificial Intelligence, 12821–12828 (AAAI, 2020).

[69] Zhang, Y. L. et al. Image super-resolution using very deep residual channel attention networks. Proceedings of the 15th European Conference on Computer Vision, 294–310 (Springer, 2018).

[70] Zhao, L. M. et al. On the connection of deep fusion to ensembling. http://arxiv.org/abs/1611.07718 (2016).

[71] Shi, Z. et al. HSCNN+: advanced CNN-based hyperspectral recovery from RGB images. Proceedings of 2018 IEEE/CVF Conference on Computer Vision and Pattern Recognition Workshops, 1052–10528 (IEEE, 2018).

[72] Peng, H., Chen, X. M. & Zhao, J. Residual pixel attention network for spectral reconstruction from RGB images. Proceedings of 2020 IEEE/CVF Conference on Computer Vision and Pattern Recognition Workshops, 2012–2020 (IEEE, 2020).

[73] Zhao, Y. Z. et al. Hierarchical regression network for spectral reconstruction from RGB images. Proceedings of 2020 IEEE/CVF Conference on Computer Vision and Pattern Recognition Workshops, 1695–1704 (IEEE, 2020).

[74] Ronneberger, O., Fischer, P. & Brox, T. U-Net: convolutional networks for biomedical image segmentation. Proceedings of the 18th International Conference on Medical Image Computing and Computer-Assisted Intervention, 234–241 (Springer, 2015).

[75] Shi, W. Z. et al. Real-time single image and video super-resolution using an efficient sub-pixel convolutional neural network. Proceedings of 2016 IEEE Conference on Computer Vision and Pattern Recognition, 1874–1883 (IEEE, 2016).

[76] Gatys L, Ecker A, Bethge M (2016). A neural algorithm of artistic style. J. Vis..

[77] Johnson, J., Alahi, A. & Fei-Fei, L. Perceptual losses for real-time style transfer and super-resolution. Proceedings of the 14th European Conference on Computer Vision, 694–711 (Springer, 2016).

[78] Windrim L (2018). A physics-based deep learning approach to shadow invariant representations of hyperspectral images. IEEE Trans. Image Proc..

[79] Kaya, B., Can, Y. B. & Timofte, R. Towards spectral estimation from a single RGB image in the wild. Proceedings of 2019 IEEE/CVF International Conference on Computer Vision Workshop, 3546–3555 (IEEE, 2019).

[80] Li, J. J. et al. Adaptive weighted attention network with camera spectral sensitivity prior for spectral reconstruction from RGB images. Proceedings of 2020 IEEE/CVF Conference on Computer Vision and Pattern Recognition Workshops, 1894–1903 (IEEE, 2020).

[81] Schanda, J. CIE 1931 and 1964 standard colorimetric observers: history, data, and recent assessments. in Encyclopedia of Color Science and Technology (ed. Luo, M. R.), 125–129 (Springer, 2016).

[82] Lin YT, Finlayson GD (2020). Physically plausible spectral reconstruction. Sensors.

[83] Nie, S. J. et al. Deeply learned filter response functions for hyperspectral reconstruction. Proceedings of 2018 IEEE/CVF Conference on Computer Vision and Pattern Recognition, 4767–4776 (IEEE, 2018).

[84] Song HY (2021). Deep-learned broadband encoding stochastic filters for computational spectroscopic instruments. Adv. Theory Simul..

[85] Zhang WY (2021). Deeply learned broadband encoding stochastic hyperspectral imaging. Light: Sci. Appl..

[86] Han X (2021). Inverse design of metasurface optical filters using deep neural network with high degrees of freedom. InfoMat.

[87] Oiknine Y (2019). Compressive sensing hyperspectral imaging by spectral multiplexing with liquid crystal. J. Imag..

[88] Gedalin D, Oiknine Y, Stern A (2019). DeepCubeNet: reconstruction of spectrally compressive sensed hyperspectral images with deep neural networks. Opt. Express.

[89] Yasuma F (2010). Generalized assorted pixel camera: postcapture control of resolution, dynamic range, and spectrum. IEEE Trans. Image Proc..

[90] Hyperspectral & Color Imaging. Hyperspectral Images Database. https://sites.google.com/site/hyperspectralcolorimaging/dataset.

[91] Habili, N., Oorloff, J. & Wei, R. Scyllarus hyperspectral dataset. https://scyllarus.data61.csiro.au/data/.

[92] Yan LB (2020). Reconstruction of hyperspectral data from RGB images with prior category information. IEEE Trans. Comput. Imag..

[93] Shrestha R (2014). Quality evaluation in spectral imaging-quality factors and metrics. J. Int. Colour Assoc..

[94] Redmon, J. et al. You only look once: unified, real-time object detection. Proceedings of 2016 IEEE Conference on Computer Vision and Pattern Recognition, 779–788 (IEEE, 2016).

[95] Yan LB (2021). Object detection in hyperspectral images. IEEE Signal Proc. Lett..

[96] Zoph, B. & Le, Q. V. Neural architecture search with reinforcement learning. https://arxiv.org/abs/1611.01578 (2016).

[97] Han, K. et al. A survey on vision transformer. https://arxiv.org/abs/2012.12556 (2020). 10.1109/TPAMI.2022.3152247 35180075

[98] Stiebel, T. et al. Reconstructing spectral images from RGB-images using a convolutional neural network. Proceedings of 2018 IEEE/CVF Conference on Computer Vision and Pattern Recognition Workshops, 948–953 (IEEE, 2018).

[99] Can, Y. B. & Timofte, R. An efficient CNN for spectral reconstruction from RGB images. https://arxiv.org/abs/1804.04647 (2018).

[100] Koundinya, S. et al. 2D-3D CNN based architectures for spectral reconstruction from RGB images. Proceedings of 2018 IEEE/CVF Conference on Computer Vision and Pattern Recognition Workshops, 957–9577 (IEEE, 2018).

[101] Ji SW (2013). 3D convolutional neural networks for human action recognition. IEEE Trans. Pattern Anal. Mach. Intel..

[102] Banerjee, A. & Palrecha, A. MXR-U-nets for real time hyperspectral reconstruction. https://arxiv.org/abs/2004.07003 (2020).

[103] He, T. et al. Bag of tricks for image classification with convolutional neural networks. Proceedings of 2019 IEEE/CVF Conference on Computer Vision and Pattern Recognition, 558–567 (IEEE, 2019).

[104] Misra, D. Mish: a self regularized non-monotonic activation function. The 31st British Machine Vision Virtual Conference: online (2020).

[105] Wang WJ, Wang JW (2021). Double ghost convolution attention mechanism network: a framework for hyperspectral reconstruction of a single RGB image. Sensors.

[106] Han, K. et al. GhostNet: more features from cheap operations. Proceedings of 2020 IEEE/CVF Conference on Computer Vision and Pattern Recognition, 1577–1586 (IEEE, 2020).

[107] Wang, X. L. et al. Non-local neural networks. Proceedings of 2018 IEEE/CVF Conference on Computer Vision and Pattern Recognition, 7794–7803 (IEEE, 2018).

